# Transcriptome-wide m6A landscape across diverse porcine tissues revealed by nanopore direct RNA sequencing

**DOI:** 10.3389/fgene.2025.1725608

**Published:** 2025-11-21

**Authors:** Yaya Liao, Zifeng Ding, Xiaoyun Chen, Chao Yin, Bin Yang, Qiang Yang, Yuyun Xing

**Affiliations:** National Key Laboratory for Pig Genetic Improvement and Germplasm Innovation, Ministry of Science and Technology, Jiangxi Agricultural University, Nanchang, China

**Keywords:** pig, direct RNA sequencing, novel transcripts, m6A modification, alternative splicing

## Abstract

N6-methyladenosine (m6A) is the most prevalent internal RNA modification in eukaryotic messenger RNA (mRNA). Pigs are valuable not only as a source of meat protein but also as ideal animal models for studying human diseases. To date, m6A has not been systematically mapped in a body-wide survey of porcine tissues. In this study, we used direct RNA sequencing data of 39 sow samples (from 23 tissues) and 7 fetal samples (from 7 tissues) to identify m6A modifications and alternative splicing (AS) events. In total, we identified 60,823 transcripts, including 27,823 novel isoforms. The mean poly(A) tail length varied markedly among tissues, ranging from 48 to 101 nt. A total of 343,951 m6A sites were identified, with sow and fetal samples averaging 80,336 and 92,476 sites, respectively. The number of m6A sites varied across different samples, ranging from 27,830 to 118,042. The brain samples displayed the most pronounced region-specific m6A pattern; different anatomical locations within the same tissue exhibited high m6A heterogeneity. Overall, m6A methylation levels were positively correlated with transcript expression levels; integrative analyses further supported an association between m6A modification and AS. Our findings provide novel insights that enhance our understanding of the regulatory complexity of the transcriptome and epitranscriptome in pigs.

## 1 Introduction

RNA modifications play crucial roles in a wide array of cellular functions by dynamically regulating RNA stability, splicing, processing, translation and metabolism (Bu et al., 2024). To date, more than 170 types of RNA modifications have been discovered, among which, N6-methyladenosine (m6A) is the most abundant and widespread internal RNA modification in eukaryotic messenger RNA (mRNA) (Huo et al., 2020). Extensive studies have demonstrated that m6A plays critical roles in various biological processes including development (Frye et al., 2018), reproduction (Wang et al., 2024), aging (Sun et al., 2022), metabolism (Liu et al., 2024), cancers and non-cancer diseases (Boulias and Greer, 2023), as well as in mRNA metabolism including alternative splicing (AS) (Wang et al., 2022).

Since the pioneering development of the initial m6A sequencing technologies in 2012 (Dominissini et al., 2012), a large number of high-throughput approaches have been established for revealing the transcriptome-wide distribution of m6A RNA modification, based on the next-generation and third-generation sequencing technologies (Hartstock and Rentmeister, 2019). Next-generation sequencing-based m6A detection methods include both widely used antibody-dependent approaches (e.g., MeRIP-seq, miCLIP) and enzyme-based, antibody-independent techniques (e.g., Mazter-seq, m6A-SAC-seq) (Hartstock and Rentmeister, 2019; Yang et al., 2024). However, these approaches may encounter challenges such as limited antibody specificity, motif preference of endoribonuclease, and variations in labeling efficiency (Zhao et al., 2020). A third-generation sequencing technology, direct RNA sequencing (DRS) developed by Oxford Nanopore Technologies (ONT), has the potential to overcome many limitations inherent in the next-generation sequencing-based m6A mapping methods. DRS bypasses the need for cDNA synthesis by reverse transcription and PCR amplification, thus provides a more accurate and faithful representation of the native RNA molecules (Workman et al., 2019). To date, DRS technology has been widely used to detect the m6A post-transcriptional modification in various organisms including yeast, Arabidopsis, rice, maize, human, and mouse (Hendra et al., 2022; He et al., 2024; Jiang et al., 2025; Xie et al., 2025). These studies not only confirmed the accuracy and reliability of DRS technology but also further underscored its unparalleled strengths in analyzing complex transcriptomes.

Pigs are not only an important source of meat for humans, but are also considered an ideal animal model for studying human development and diseases, and are promising organ donors for xenotransplantation (Lunney et al., 2021). To date, a few studies have been conducted to elucidate m6A sites and to uncover the underlying epigenetic mechanisms in pigs. However, the vast majority of these studies employed MeRIP-seq method and are limited to a single tissue type, such as muscle (Dou et al., 2023; Gong et al., 2023), liver (He et al., 2017), or ovary (Li et al., 2023). Thus, the dynamics of m6A among different porcine tissues remain largely unknown. We believe that in-depth exploration and analysis of m6A sites across various porcine tissues are of significant importance for pig epigenetic studies. In this study, we used ONT DRS technology to systematically profile full-length transcriptomes from 39 samples representing 24 tissues of two Large-White sows, together with 7 additional samples from fetal tissues of one of these sows. We successfully constructed the high-resolution map of m6A modification sites in various porcine tissues and further explored the AS events across these tissues.

## 2 Methods

### 2.1 Animals and sample collection

Two Large White sows at 110 days of gestation from the same pig farm were initially anesthetized with Zoletil-50 (a combination of 25 mg/mL tiletamine and 25 mg/mL Zolazepam) via intramuscular injection at a dose of 5 mg/kg body weight. General anesthesia was maintained with 1%–2% isoflurane gas. After all fetuses were removed, the sows were euthanized by intravenous injection of potassium chloride solution (100 mg/kg body weight), and tissue samples were immediately collected. A total of 39 samples were obtained from two sows with identical genetic background and similar age, comprising 33 samples from one sow and 6 samples from the second, collected within the same time window. The samples from the first sow covered a wide range of anatomical regions, including the adipose, cardiovascular, digestive, reproductive, urinary, lymphatic, muscular, respiratory, and integumentary systems. The second sow provided these six samples: cerebellum, temporal lobe, occipital lobe, brainstem, tongue, and pancreas, since the collection of these tissues was unsuccessful in the first sow. In addition, a total of 7 samples were collected from one male fetus of the first sow. These 7 samples included the cardiac ventricle, cerebellum, right outer lobe of the liver, kidney, thymus, longissimus dorsi muscle, and medial segment of the middle lobe of lung (Supplementary Figure S1). All collected samples were promptly frozen in liquid nitrogen and subsequently stored at −80 °C for further experiments.

### 2.2 RNA extraction, ONT DRS and illumina sequencing

Total RNA from the tissues mentioned above was isolated and purified using TRIzol reagent (Invitrogen, Carlsbad, CA, United States) according to the manufacturer’s instructions. The RNA quality and concentration were assessed using a NanoDrop One spectrophotometer (Thermo Fisher Scientific) for purity (A260/A280), a Qubit 3.0 fluorometer (Life Technologies) for accurate quantification, and agarose gel electrophoresis for integrity evaluation. Approximately 25 µg of qualified RNA from each sample was used to enrich poly(A) mRNA using the NEBNext Poly(A) mRNA Magnetic Isolation Module (NEB, E7490S), following the manufacturer’s protocol. The DRS libraries were constructed using ONT SQK-RNA002 kit (ONT, Oxford, United Kingdom) according to the manufacturer’s instructions. Then the constructed libraries were loaded onto the ONT R9.4 flow cells and sequenced on the PromethION sequencer (ONT, Oxford, United Kingdom).

For Illumina sequencing, 2 µg of total RNA for each sample was used to construct sequencing libraries using NEBNext® Ultra™ RNA Library Prep Kit for Illumina® (NEB, E7530L) following the manufacturer’s instructions. The prepared libraries were then subjected to transcriptome sequencing on an Illumina NovaSeq platform using paired-end 150 nt reads (Benagen, Wuhan, China).

### 2.3 Base-calling, filtering and mapping

The raw sequencing data, comprising continuous current trajectories generated by the PromethION sequencer, were stored in the FAST5 format. These data were then base-called using the default parameters of the GUPPY software (version 3.2.6). To enhance data quality, Nanofilt (version 2.6.0) (De Coster et al., 2018) was employed to filter out low-quality reads (quality score <7), short reads (<50 nt), adapter-contaminated reads, and unknown reads. The filtered reads were further corrected using FCLMR2 (version 0.1.2) (Mak et al., 2023) in conjunction with next-generation RNA-Seq data to generate clean reads. These clean reads were aligned to the porcine reference genome (Sscrofa 11.1) using minimap2 (version 2.17) (Li, 2018). The aligned sequences were processed to obtain consensus and non-redundant sequences using Flair (version 1.4.0) (Tang et al., 2020). Subsequently, StringTie2 (version 2.1.5) (Kovaka et al., 2019) was used to merge the aligned sequences, generating a novel reference transcript file for the porcine genome. Novel transcripts from ONT DRS were identified using GffCompare (version 0.12.6) (Pertea and Pertea, 2020) with parameter -R -C -K. The tool compares exon–intron structures of assembled transcripts against the reference annotation and assigns class codes; transcripts with class codes o, j, x, i, and u were considered novel and retained for downstream analyses. Finally, the read coverage was visualized using the Integrative Genomics Viewer (IGV) tools (Robinson et al., 2011).

### 2.4 Expression profiling of next-generation sequencing datasets

Illumina sequencing reads were trimmed and subjected to quality control using Fastp (version 0.23.2) (Chen et al., 2018). The clean reads were then aligned to the porcine reference genome (Sscrofa 11.1) using Hisat2 (version 2.1.0) (Kim et al., 2019). Subsequently, StringTie2 (version 2.1.5) (Kovaka et al., 2019) was employed to assemble the transcripts and quantify the expression levels of genes and transcripts.

### 2.5 Poly(A) tail length estimation

The poly(A) tail length of all reads was determined by the raw signal according to Nanopolish (Workman et al., 2019). The poly(A) length of reads that passed through Nanopolish was retained for further studies.

### 2.6 Confirmation of novel transcripts via PCR

Six randomly selected novel transcripts were PCR amplified from cDNA for sequencing (primers are listed in Supplementary Table S1). The PCR products were purified using the Agarose Gel DNA Extraction Kit (DP219, Tiangen) and used for TA cloning-based sequencing. The sequencing data generated were assembled and analyzed using the SeqMan Ultra sequence viewer in DNASTAR Lasergene 17 software.

### 2.7 Identification and analysis of m6A modification sites

Briefly, the multi_to_single_fast5 tool was used to demultiplex multi-read FAST5 files into individual single-read files. Then the re-squiggle function of Tombo (version 1.5.1, https://github.com/nanoporetech/tombo) was employed for alignment, followed by the application of the MINES (Lorenz et al., 2020) tool to identify all regions containing DRACH motifs (D = A/G/U, R = A/G, H = A/C/U). New regions were generated by extending 10 base pairs on each side of the “A” base within the DRACH motif. High-confidence m6A sites were defined using stringent thresholds of read coverage >5 and m6A fraction >0.5, ensuring reliable detection of substantially methylated sites. The m6A level of each site was estimated by multiplying the m6A coverage with the m6A fraction. Pairwise Pearson correlations were then computed across samples, and the correlation heatmap was generated using the ComplexHeatmap package (version.2.8.0). MetaPlotR (Olarerin-George and Jaffrey, 2017) was used to visualize the density distribution of m6A modifications across the 5′ UTR, CDS, and 3′ UTR of mRNA transcripts. Each segment was normalized based on the length of the transcript.

### 2.8 Functional enrichment analysis

GO-BP enrichment results for the m6A-methylated genes were obtained using the DAVID online platform (https://davidbioinformatics.nih.gov/) and functional annotations were assigned with the Gene Ontology database.

### 2.9 Detection of AS events

SUPPA2 software (https://github.com/comprna/SUPPA) was applied to analyze the AS types including SE (skipped exon), MX (mutually exclusive exons), A5 (alternative 5′ splice site), A3 (alternative 3′ splice site), RI (retained intron), AF (alternative first exon), and AL (alternative last exon), across all the detected samples.

## 3 Results

### 3.1 DRS data set

**FIGURE 1:**
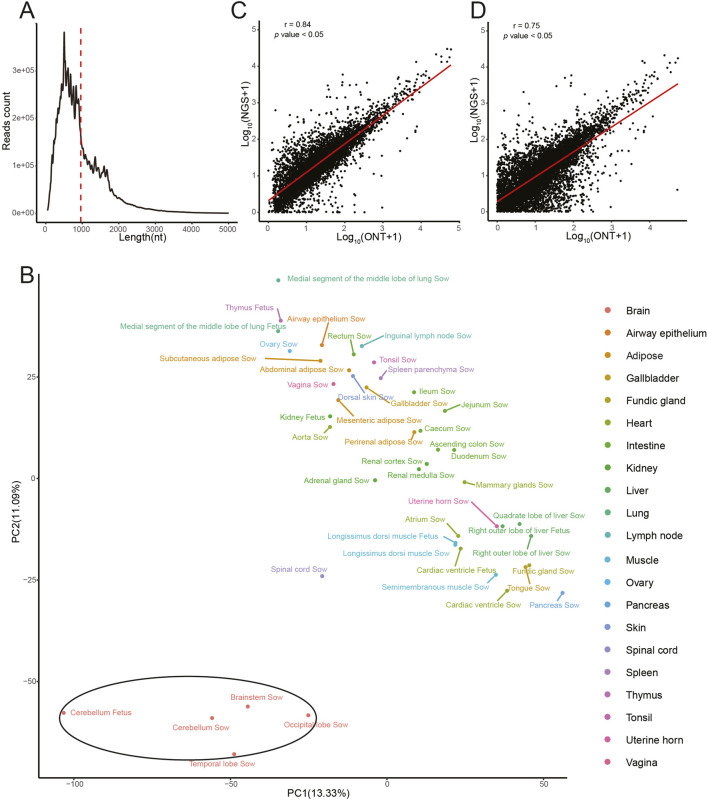
Summary of the direct RNA sequencing data. **(A)** The distribution of reads lengths across all sequenced samples. Red dashed line indicates the mean read length. The X-axis is truncated at 5,000 nt. **(B)** PCA was performed on transcriptome data across tissues. The plot displays the first two principal components (PC1 = 13.33%, PC2 = 11.09%) calculated from transcripts with TPM >20 in at least three samples. Each point represents a sample, colored by tissue type. Pearson correlation of gene **(C)** and transcript **(D)** expression levels in spleen parenchyma of sow based on DRS and Illumina sequencing. The Pearson’s r values were shown in the upper left corner of the figure.

A total of 46 sequencing libraries were constructed, including 39 libraries from sow samples (covering 23 tissues) and 7 libraries from fetal samples (covering 7 tissues) (Supplementary Figure S1). These libraries were loaded onto ONT R9.4 flow cells. After adapter trimming and reads filtration, the clean read counts obtained for these 46 detected samples ranged from 3,735,465 to 11, 815, 120. The median read length across all samples was 775 nt, while the mean read length was 960 nt (ranged from 677.6 to 1226.1 nt; Figure 1A; Supplementary Table S2), and the reads length distribution of the 46 sequenced samples was similar (Supplementary Figure S2). Alignment of the sequenced data with reference sequences for all detected samples exceeded 96%. To explore the transcriptome-wide expression relationships among samples, we first filtered the data for highly expressed transcripts (TPM >20 in at least three samples) and then performed Principal Component Analysis (PCA) to visualize the resulting sample-to-sample similarities. The cerebellum, brainstem, temporal lobe, and occipital lobe clustered together in the PCA and were distinctly separated from other tissues, highlighting the unique expression patterns of brain tissues (Figure 1B). Pearson correlation coefficient was calculated by using the DRS and Illumina sequencing data. The mean correlation coefficients were 0.86 for genes and 0.77 for transcripts, respectively (Supplementary Table S3). For example, in the spleen parenchyma of a sow, the correlation coefficients were 0.84 for expressed genes and 0.75 for transcripts (Figures 1C,D). Our findings revealed the correlations of the expressed genes across all detected samples were higher than those of the corresponding transcripts, likely due to the presence of multiple transcripts originated from a single gene. The significant correlations observed across all detected samples underscored the precision of DRS technology.

### 3.2 Examination of novel transcripts and poly(A) tail length variations

**FIGURE 2:**
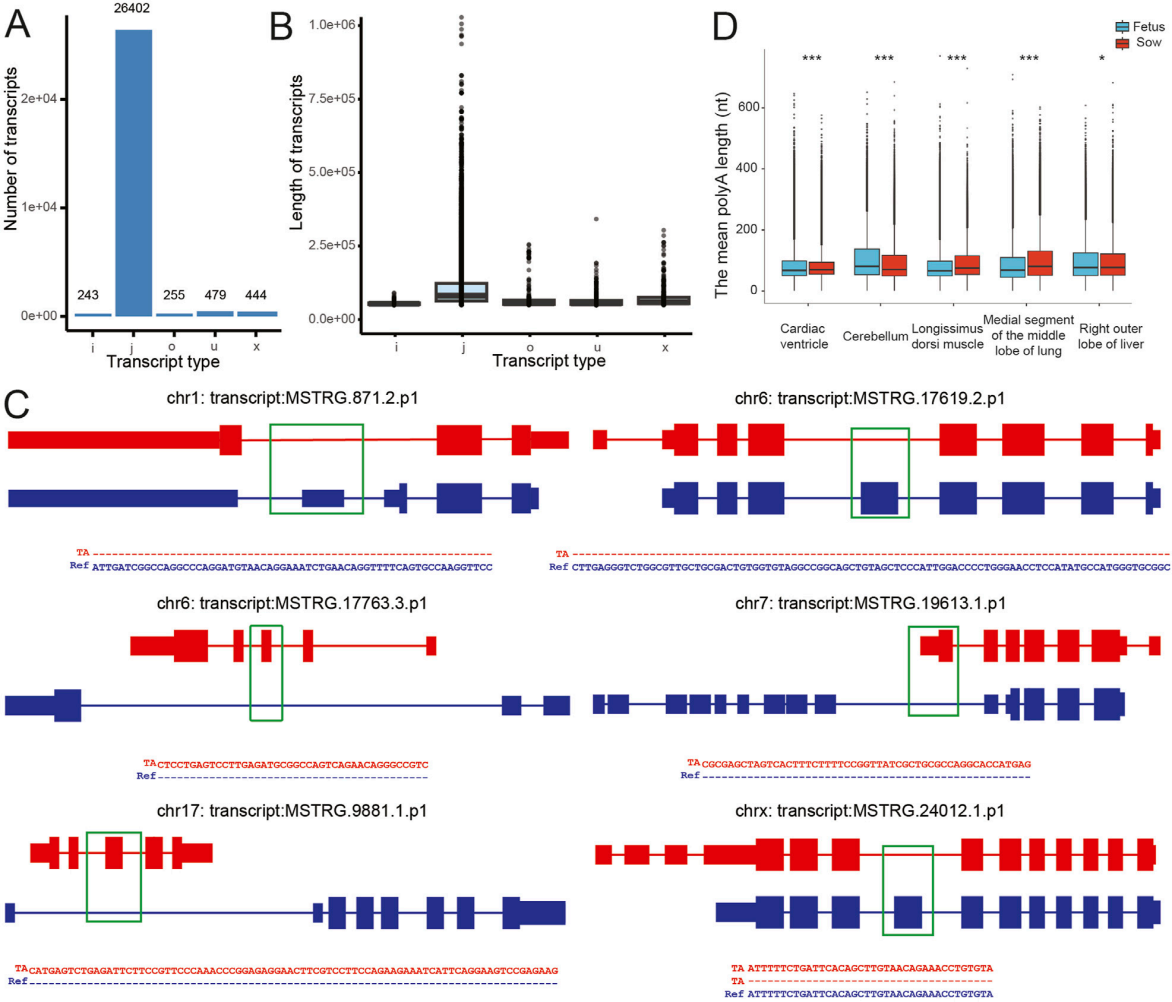
Identification of novel transcripts. **(A)** The number of novel transcripts identified based on Gffcompare. Class code. i, fully contained within a reference intron; j, multi-exon with at least one junction match; o, other same strand overlapping with reference exons; u, none of the above (unknown, intergenic); x, exonic overlapping on the opposite strand. **(B)** The length distributions of different types of novel transcripts. **(C)** Verification of the randomly selected novel transcripts identified by DRS. ref: reference sequence; TA: The sequences obtained from TA cloning-based sequencing. **(D)** Comparison of mean poly(A) lengths of 5 common tissues between the sows and fetus. The Wilcoxon test was used to compare the length of poly(A) between sows and fetus in the same tissue. *: *P* < 0.05; ***: *P* < 0.001.

A total of 60,823 transcripts were identified across the detected samples. The number of expressed genes varied from 11,610 in the sow pancreas to 17,209 in the sow ileum, and the number of transcripts ranged from 14,200 in the sow pancreas to 28,878 in the sow brainstem (Supplementary Table S2). Among them, 27,823 novel transcripts (isoforms) were identified and were not present in the reference genome (Figure 2A), with counts per sample ranging from 2,448 (sow pancreas) to 8,558 (sow cerebellum) (Supplementary Figure S3A). These novel transcripts are divided into 5 types according to the GffCompare software, and the numbers of o, j, x, i and u types of novel transcripts were 255, 26,402, 444, 243 and 479, respectively. These novel transcripts exhibited different length distributions across different categories (Figure 2B). To validate the accuracy of novel transcript detection, 6 identified isoforms were randomly selected for PCR amplification and subsequent TA cloning-based sequencing. The sequencing data confirmed the reliability of the novel transcript detection (Figure 2C; Supplementary Figure S3B). It should be noted that 2 transcripts of MSTRG.240.12.1. p1 were identified through PCR and TA cloning-sequencing. One of these transcripts corresponds to a known annotated transcript (the major transcript), while the other represents a newly identified transcript (the minor transcript).

The poly(A) tail plays vital roles in mRNA stability and translation regulation. In this study, we calculated the length of poly(A) tail of transcripts across all detected samples. The data revealed that the median poly(A) tail length was 70 nt, varying from 34.78 in sow pancreas to 82.06 in the quadrate lobe of liver of the sow (Supplementary Figure S4), while the mean poly(A) tail length ranged from 48.12 in sow pancreas to 101.04 in fetal cerebellum (Supplementary Table S4). Notably, significant differences in poly(A) tail lengths were found among all these 5 counterpart-tissue samples between sow and fetus, though the media and mean poly(A) tail lengths in cardiac ventricle and right outer lobe of liver were close. The mean poly(A) tail lengths in longissimus dorsi muscle and medial segment of the middle lobe of lung were significantly higher in the sow than those in the fetus; however, the mean poly(A) tail length in cerebellum was significantly lower in the sow than in the fetus (Figure 2D; Supplementary Table S4).

### 3.3 Distribution of m6A modification across different tissues, mRNA regions and chromosomes

**FIGURE 3:**
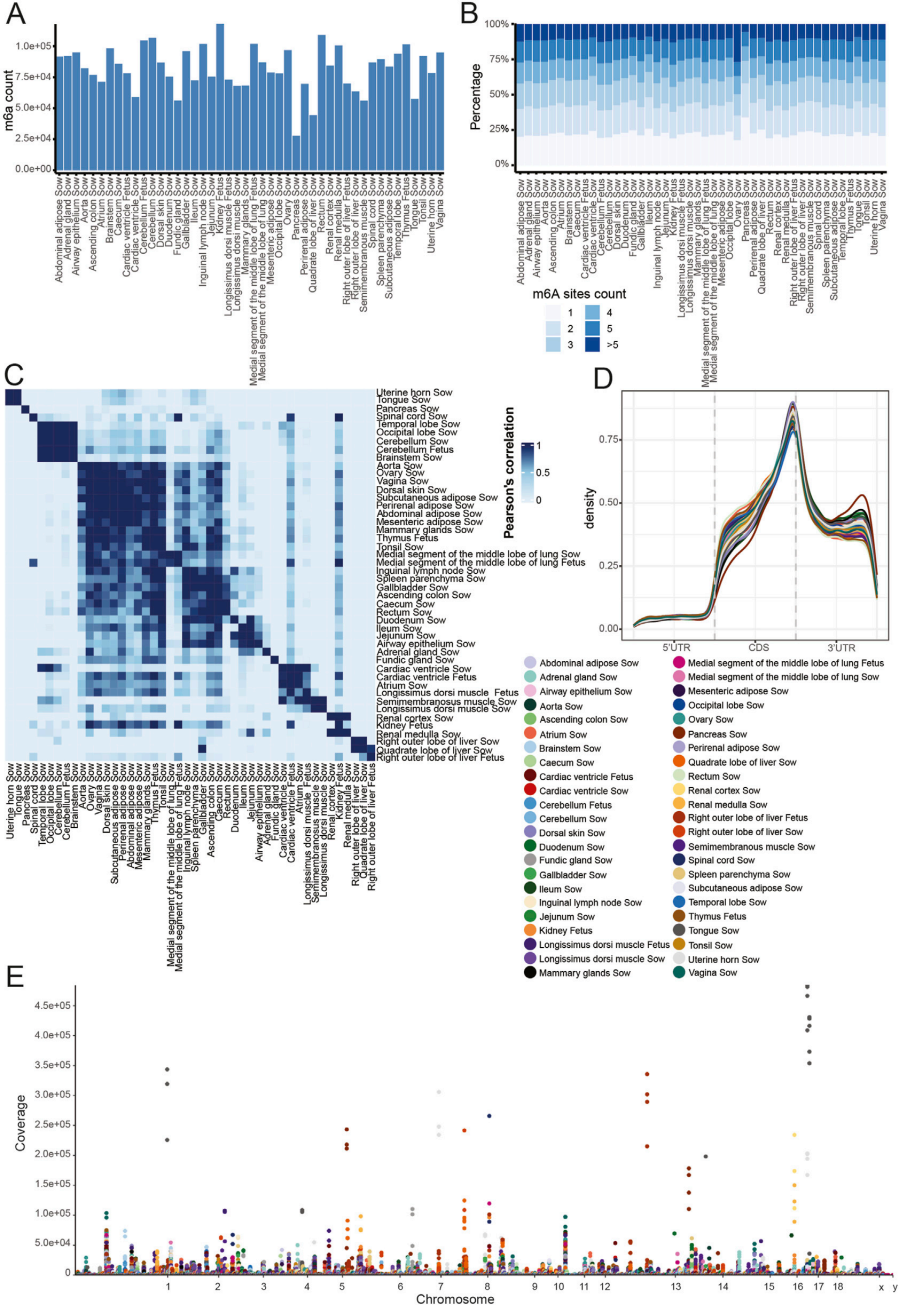
The distribution of m6A modification across different samples and mRNA regions. **(A)** The number of m6A modification sites in different samples. **(B)** The ratio of transcripts with different numbers of m6A modification sites. **(C)** The Pearson’s correlation of m6As between different tissues. The m6A level of each site was estimated by multiplying the m6A coverage with the m6A fraction. **(D)** Metaplot of m6A modification density distribution in the 5′ UTR, CDS and 3′ UTR of mRNA transcripts from different samples. Each segment was normalized based on the mean length defined in the reference sequence annotation. **(E)** Distribution of m6A coverage across different chromosomes in various porcine tissues. Each curve in **(D)** or dot in **(E)** represents the m6A distribution in one sample, with different colours indicating different samples from sow and fetus.

Using the Tombo and MINES tools, we identified a total of 343,951 m6A modifications across the sow and fetus samples. Fetal samples harbored a significantly higher number of m6A modifications compared with corresponding sow samples, with mean counts of 92,476 and 80,336 sites, respectively (Figure 3A). There were 8 samples in which the number of m6A sites exceeded 100,000; these included the cerebellum, renal medulla, rectum, and inguinal lymph node from sows, as well as the cerebellum, thymus, medial segment of the middle lobe of lung, and kidney from the fetus. Moreover, more than 75% of the transcripts were modified by m6A in each sample, except for quadrate lobe of liver in the sow (Supplementary Figure S5). To explore the relationship between m6A methylation and transcripts expression, we compared the expression levels of transcripts with and without m6A modifications. The expression levels of transcripts carrying m6A sites were significantly higher than those lacking such modifications (Supplementary Figure S6A). In addition, transcript expression levels increased overall with the number of m6A sites (Supplementary Figure S6B). Among the transcripts containing m6A sites, more than 50% had 2 or more m6A sites, and approximately 10% of the transcripts had 5 or more m6A sites (Figure 3B). To assess the correlation of m6A modifications across different tissues, we employed the Complex Heatmap software to conduct clustering analysis. The data revealed a high degree of correlation among m6A sites within the same tissue types. For example, the m6A modification sites in different brain tissues, such as the temporal lobe, occipital lobe, cerebellum, and brainstem, were highly correlated and formed a distinct clustering group (Figure 3C). Similar findings were also observed in intestinal, muscle, kidney and liver tissues.

Next, we employed MetaplotR package to characterize the m6A distribution along gene body and UTRs. The data indicated that m6A modifications were predominantly enriched in the CDS and 3′ UTR, while the presence of m6A in the 5′ UTR was significantly lower (Figure 3D). We further examined the m6A coverage across all the chromosomes in the detected samples. The results indicated that m6A sites were widely distributed across all chromosomes, whereas significant differences in the coverage of m6A among different samples were observed (Figure 3E). Moreover, we compiled a list of the top 10 m6A sites with the highest levels of coverage across all chromosomes, along with their corresponding samples and genes. Most of these genes are associated with reported functions in the corresponding tissues (Supplementary Table S5).

### 3.4 Intra-tissue regional m6A heterogeneity versus ubiquitous sites

In this study, we collected ≥3 anatomically distinct samples from brain, heart, adipose, and intestine tissues. These 4 tissues yielded a total of 162,216, 119,617, 145,771, and 172,456 m6A sites, respectively. Among these, only 27.14%, 26.23%, 22.83%, and 16.16% of the m6A sites were shared among the different regions within the same tissue (Supplementary Figure S7). These findings underscore pronounced intra-tissue heterogeneity of m6A modification and suggest that m6A may contribute to the establishment of region-specific functional identities and microenvironmental adaptation.

**FIGURE 4:**
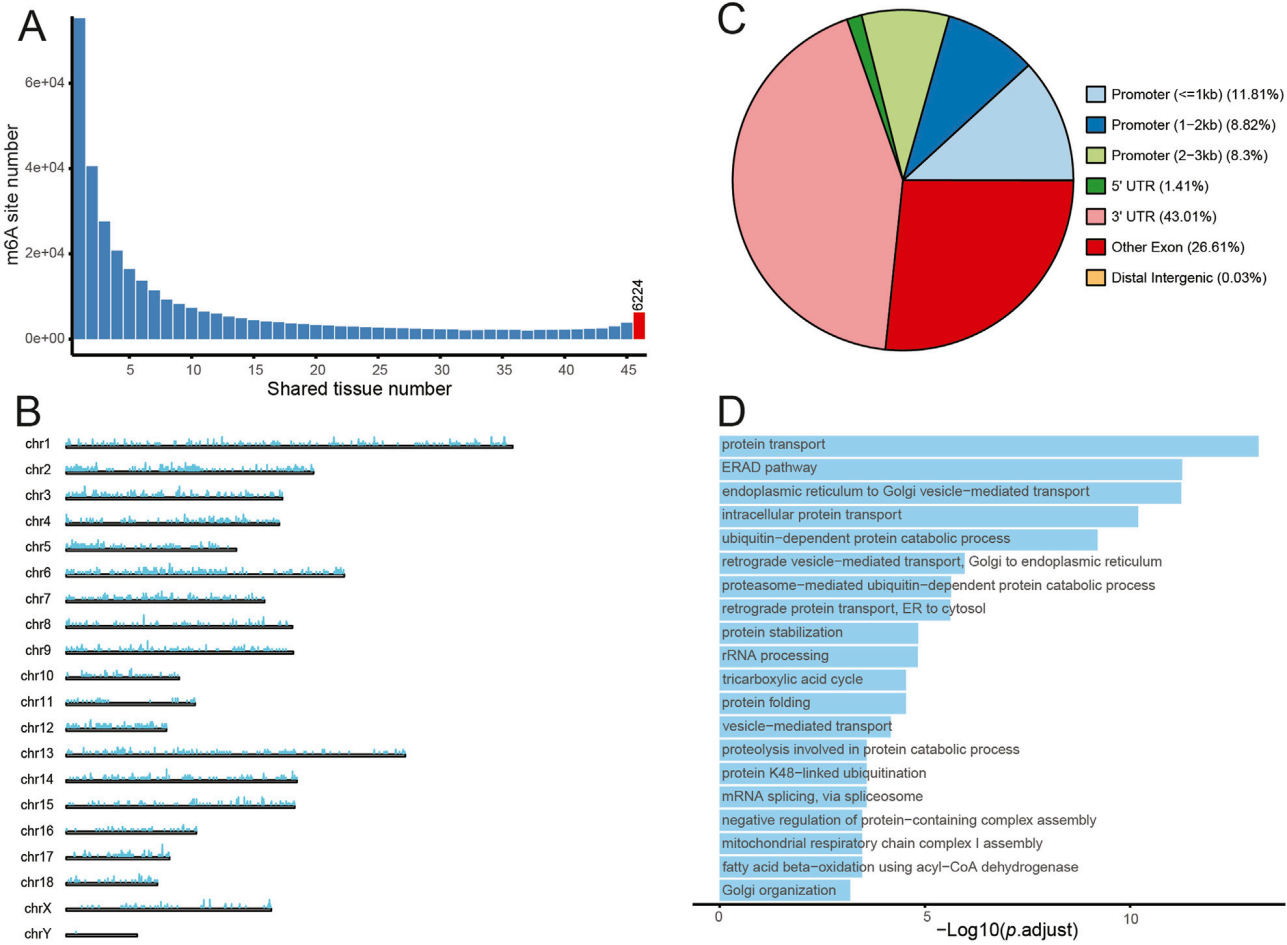
The distribution of ubiquitous m6A sites. **(A)** The distribution of m6A counts in one or more samples. **(B)** The distribution of ubiquitous m6A sites across chromosomes. **(C)** Pie chart of ubiquitous m6A sites within promoter, 5′UTR, 3′UTR, other exon and distal intergenic regions. **(D)** GO-BP enrichment results for genes containing ubiquitous m6A modification.

Next, we analysed the distribution of all m6A sites across all 46 samples derived from the sow and fetus. The findings revealed that 75,186 m6A sites were present exclusively in a single sample, whereas 6,224 m6A sites were identified across all 46 samples (Figure 4A). These 6,224 ubiquitous m6A sites were distributed in 2,822 genes, and visualization map showed that SSC 17, 12, 2, 6, and 5 exhibited relatively higher ubiquitous m6A densities (Figure 4B). In addition, we investigated the distribution patterns of ubiquitous m6A sites across mRNA transcripts. The data showed that nearly half of the sites are located in the 3′ UTR, approximately 27% of the sites are found in the exon regions, and less than 2% of the sites are distributed in the intergenic regions and the 5′ UTR (Figure 4C). GO-BP analysis of the 2,822 genes containing the ubiquitous m6A sites revealed that the most significantly enriched pathways were related to intracellular protein metabolism, transport and folding. These pathways included protein transport, ERAD pathway, endoplasmic reticulum to Golgi vesicle-mediated transport, intracellular protein transport, ubiquitin-dependent protein catabolic process (Figure 4D).

### 3.5 AS events

**FIGURE 5:**
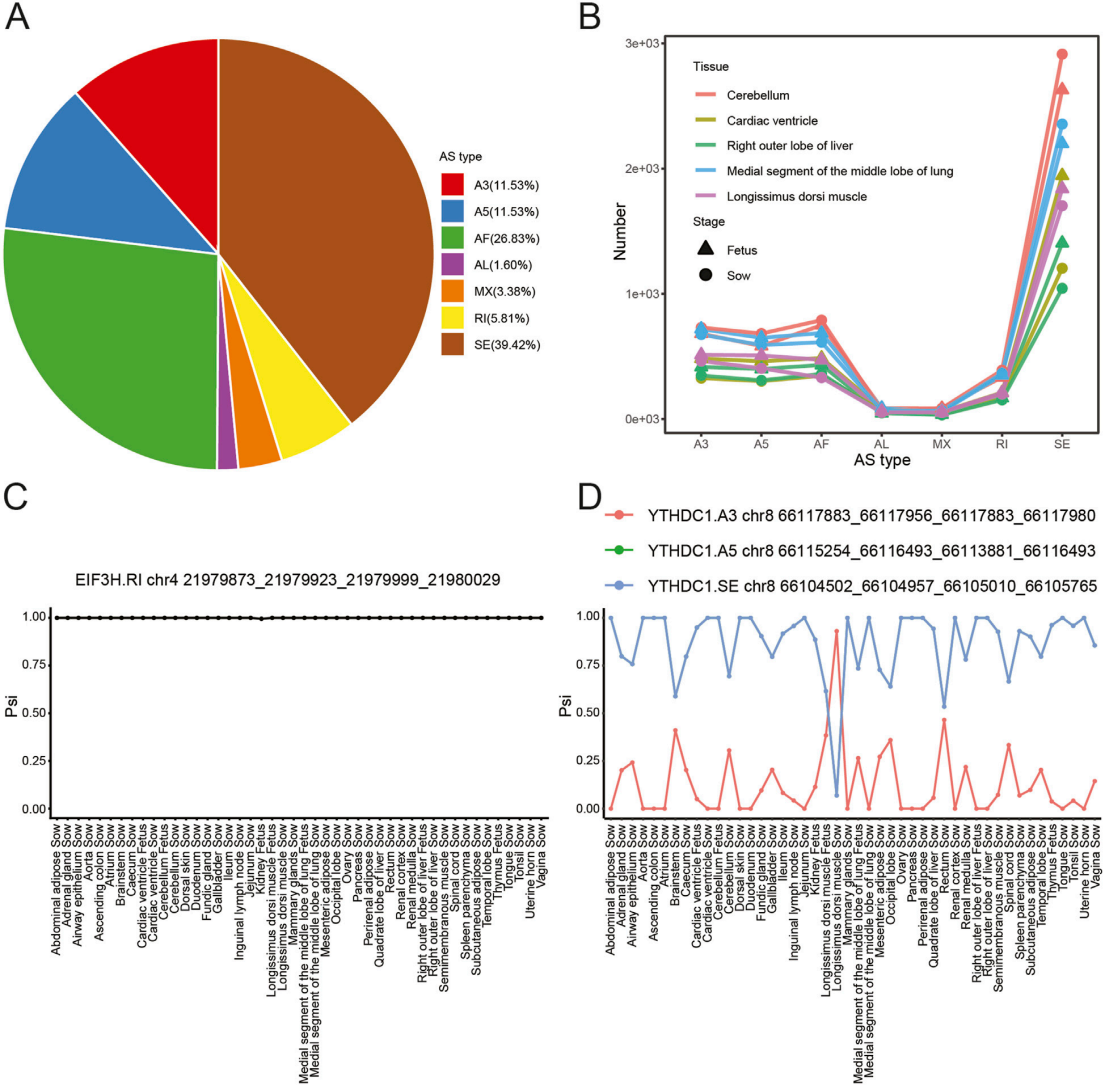
AS events in the detected samples. **(A)** Percentage distribution of AS events across 46 detected samples of sow and fetus. SE: skipped exon, AF: alternative first exon, A3: alternative 3′ splice site, A5: alternative 5′ splice site, AL: alternative last exon, MX: mutually exclusive exon, RI: retained intron. **(B)** Number of AS events in the 5 common tissues of sow and fetus. The Psi values of *EIF3H* **(C)** and *YTHDC1* **(D)** in the detected tissues of sow and fetus, respectively. Psi = splice-in/(splice-in + splice-out).

We further investigated the AS events across the detected 46 samples. The data exhibited that SE was the most prevalent type (with a proportion of 39.42%), followed by AF at 26.83%, while AL had the lowest proportion at 1.60% (Figure 5A). A similar pattern was observed when each of the 5 samples in fetus was compared with the corresponding sow samples (Figure 5B). In addition, we analyzed the Psi (percent spliced in) values of AS events for 4 m6A reader genes in all these detected samples. The findings revealed that *EIF3H* gene undergo RI across all the detected samples (Figure 5C). For the *YTHDC1* gene, the Psi values of A3 were below 0.5 in most samples, except in the sow longissimus dorsi muscle. In contrast, the Psi curves for *YTHDC1* SE and A5 overlapped, with values exceeding 0.5 in most samples, except in the sow longissimus dorsi muscle (Figure 5D). For *YTHDC2* gene, the Psi values of A5 were 1 in most samples, except in the sow ascending colon and inguinal lymph node, where the values were 0 (Supplementary Figure S8A). Similarly, the *YTHDF1* gene exhibited Psi values of 1 in all detected samples with the sole exception of the rectum, where the Psi value was 0 (Supplementary Figure S8B).

### 3.6 Association between m6A modification and AS

**FIGURE 6:**
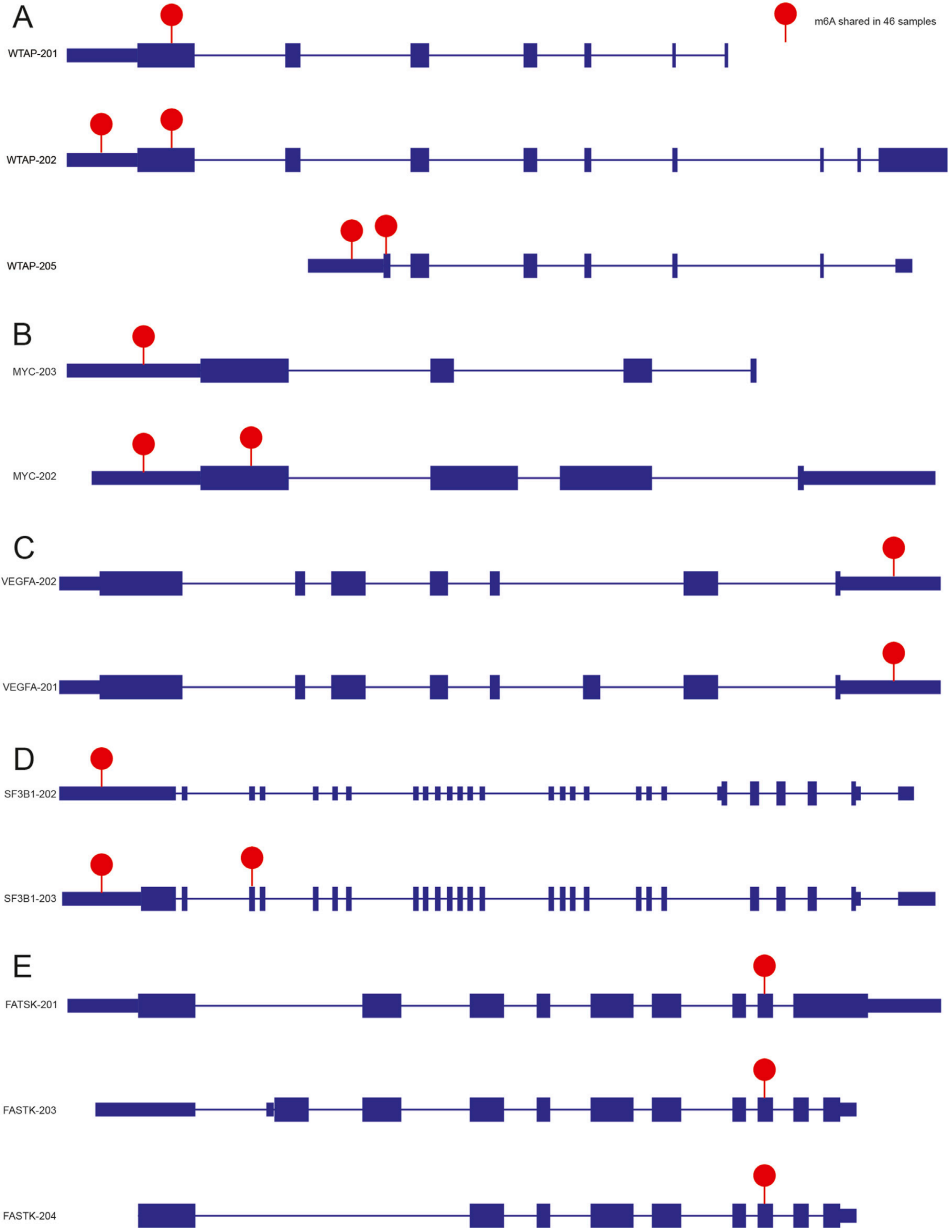
Examples for illustrating the linkage between m6A and AS. Examples of patterns for ubiquitous m6A within multiple transcripts of *WTAP* **(A)**, *MYC* **(B)**, *VEGFA* **(C)**, *SF3B1* **(D)**, and *FASTK* **(E)** genes.

To investigate the potential association between m6A modification and AS, we identified 6,224 m6A sites that were consistently shared across all 46 samples, mapping to 2,822 genes. After excluding genes with only one annotated isoform and those lacking functional annotation, 561 genes remained, collectively encoding 1,313 distinct isoforms that carried 2,272 m6A sites. Notably, the distribution of m6A sites varied among different isoforms of the same gene, suggesting a potential association between m6A deposition and isoform diversity. For instance, *WTAP* encodes three isoforms (*WTAP-201*, *WTAP-202*, and *WTAP-205*) that displayed distinct m6A profiles (Figure 6A). Comparable isoform-specific m6A distributions were also observed for *MYC* and *SF3B1*. In contrast, the m6A patterns were invariant across isoforms of VEGFA and FASTK, suggesting that m6A deposition is determined at the gene level, not in an isoform-specific manner (Figures 6B–E).

## 4 Discussion

Our DRS analysis of diverse porcine tissues revealed a highly dynamic m6A epitranscriptome, with distinct m6A profiles across different tissues, high heterogeneity within tissues, and an association between m6A and AS. This study provides the first comprehensive m6A atlas in pigs, offering foundational insights into post-transcriptional regulation during maternal-fetal development.

In the present study, PCA showed that brain samples clustered together even when they came from the second sow, whereas tongue and pancreas from the same individual grouped with the tissues from the first sow. This pattern indicates that clustering is driven by tissue-specific transcriptional profiles rather than individual effects, a conclusion further supported by the two sows being of identical genetic background, similar age, and the same gestational stage (110 days) from the same farm. While DRS accurately resolves full-length transcripts, its sensitivity for low-abundance RNAs and base-calling precision require complementary short-read sequencing (Kainth et al., 2023). Our data showed strong agreement with Illumina results, consistent with previous A549 cell studies (Chen et al., 2025). Notably, the pancreas exhibited the lowest numbers of expressed genes, transcripts, and mean poly(A) tail length among sow tissues, likely due to its inherent high RNase levels, which markedly promote RNA degradation (Jun et al., 2018; Al-Adsani et al., 2022).

In this study, we identified 60,823 porcine transcripts (27,823 novel), highlighting the incomplete annotation of the transcriptome. j-type isoforms were predominant, aligning with turtle DRS data (Zhou et al., 2022) but diverging from the u-type dominance in bovine testis (Liu et al., 2023). The high proportion (45.7%) of unannotated transcripts emphasizes the need for improved genome annotations in agricultural species (Tuggle et al., 2016) and the importance of functional validation to distinguish signal from noise (Pertea et al., 2018). We also found significant variation in poly(A) tail lengths across tissues. The median poly(A) tail length (70 nt) correlates with optimal translation, while longer tails (101 nt) in fetal cerebellum likely promote mRNA stability for neurodevelopment (Serdar et al., 2025). Marked differences of poly(A) tail length in the 5 common tissues between sow and fetus suggest developmental-stage-specific regulation by PABPs or deadenylases (Schall and Latham, 2024).

This study further provides the first comprehensive m6A atlas across 46 maternal-fetal porcine samples, revealing ubiquitous deposition and stage-specific disparities which implicate fetal-enriched RNA modification activity in driving differentiation and reprogramming (Zhao et al., 2017). By regulating mRNA splicing, export, stability and translation, m6A thereby supports rapid gene-expression switching during development (Frye et al., 2018). Facilitated by efficient RNA-processing systems, increased m6A deposition in the fetus may coordinate transcriptional networks governing growth and organogenesis. Moreover, the more open chromatin and higher transcriptional activity of fetal cells enhance recruitment of *METTL3*/*METTL14* complexes, consequently elevating global m6A levels.

Multi-modified transcripts suggest “methylation hotspots” where clustered m6A sites cooperatively regulate RNA fate (Zaccara et al., 2019). In this study, the conserved enrichment of m6A in CDS and 3′ UTRs aligns with established epitranscriptomic models (Dominissini et al., 2012). Our results revealed pronounced intra-tissue m6A heterogeneity, wherein few sites are shared across regions, a finding consistent with single-cell data (Ren et al., 2025), suggesting that this heterogeneity potentially underpins regional specialization and microenvironmental adaptation. Among all tissues, 6,224 ubiquitous m6A sites were identified, revealing fundamental principles balancing universal processes. These sites are notably enriched on chromosomes SSC 17, 12, 2, 6, and 5, suggesting epigenetic “hotspots” coordinate clustered gene regulation, akin to chromatin TADs (Dixon et al., 2012). Functionally, the enrichment of protein homeostasis pathways within these m6A-modified genes demonstrates m6A’s role in maintaining cellular proteostasis, potentially through regulating quality-control transcripts in stress responses (Engel et al., 2018).

m6A methylation dynamically regulates AS by recruiting reader proteins and modulating the spliceosome, thereby expanding isoform diversity (Zhu et al., 2023). In our study, AS analysis revealed conserved patterns: SE is the most prevalent event, consistent with its evolutionary role in reading frame preservation, followed by AF, indicative of promoter switching in tissue-specific regulation (Wang et al., 2008). RI events in genes such as *EIF3H* are ubiquitous and functionally linked to m6A-mediated ribosome loading (Mauger et al., 2016). Differential splicing of *YTHDC1* isoforms reflects its distinct regulatory roles, with muscle-specific variants underscoring tissue-dependent functional demands (Qiao et al., 2023). In contrast, constitutive splicing of *YTHDC2* and *YTHDF1* implies optimized core mRNA functions.

Moreover, our findings reveal a significant association between m6A modification and AS, supported by isoform-specific m6A distribution patterns in key regulatory genes. For instance, *WTAP*, a core methyltransferase component, not only facilitates m6A deposition but also modulates RNA splicing (Ping et al., 2014). Previous studies have shown that loss of *WTAP* perturbs alternative splicing of Sxl, tra, and other transcripts (Ortega et al., 2003), underscoring its essential role in pre-mRNA processing. Notably, m6A is markedly enriched at multi-isoform genes and alternatively spliced exons (Dominissini et al., 2012), implying that the modification fine-tunes gene expression by modulating RNA splicing. Here we report that *WTAP* itself produces multiple splice isoforms that bear m6A residues, suggesting that the modification governs *WTAP* pre-mRNA splicing and stability. Such feedback could tune m6A-complex activity and splicing patterns in a tissue- or stage-specific manner, further deepening the interplay between m6A and alternative splicing. Similarly, *MYC*, an oncogene involved in transcription and splicing (Dang, 2012), exhibits isoform-selective m6A patterning, indicating m6A-based fine-tuning of its splicing and biological output. *SF3B1*, a spliceosomal factor critical for pre-mRNA splicing (Cretu et al., 2018), also shows differential m6A deposition across isoforms, reinforcing the role of m6A in spliceosome-dependent AS regulation. In contrast, the identical m6A pattern across all isoforms of *VEGFA* and *FASTK* suggests gene-selectivity in m6A-mediated AS regulation. *VEGFA* is a key regulator of angiogenesis, and its splicing variants have distinct biological activities (Harper and Bates, 2008). The consistent m6A pattern might imply that for these genes, m6A modification is not a major determinant of isoform generation. *FASTK*, involved in mitochondrial RNA processing and splicing (Jourdain et al., 2017), also shows this gene-specific characteristic.

## 5 Conclusion

In summary, we employed Nanopore DRS to conduct the first comprehensive epitranscriptomic profiling of m6A modifications across diverse porcine tissues. We identified 60,823 transcripts, including 27,823 novel isoforms, and detected 343,951 m6A sites with distinct abundance between adult (80,336 sites) and fetal (92,476 sites) stages. Our analysis revealed substantial m6A heterogeneity across tissues, with brain regions exhibiting particularly pronounced specificity, and revealed significant associations between m6A methylation and AS regulation. This work provides new insights into the regulatory complexity of the porcine transcriptome and epitranscriptome, establishing a valuable resource for advancing genetic and disease modeling research in pigs.

## Data Availability

The datasets presented in this study can be found in the Genome Sequence Archive (GSA) at: https://ngdc.cncb.ac.cn/gsa/, with the BioProject number PRJCA048068 and accession number CRA031277.
