# Supplementary material for: Transcriptome-wide m6A landscape across diverse porcine tissues revealed by nanopore direct RNA sequencing
Source: Front Genet. 2025 Nov 21;16:1725608. doi: 10.3389/fgene.2025.1725608 (PMC12678253; doi:10.3389/fgene.2025.1725608)
Supplement: Supplementary file 1 [file DataSheet1.doc]

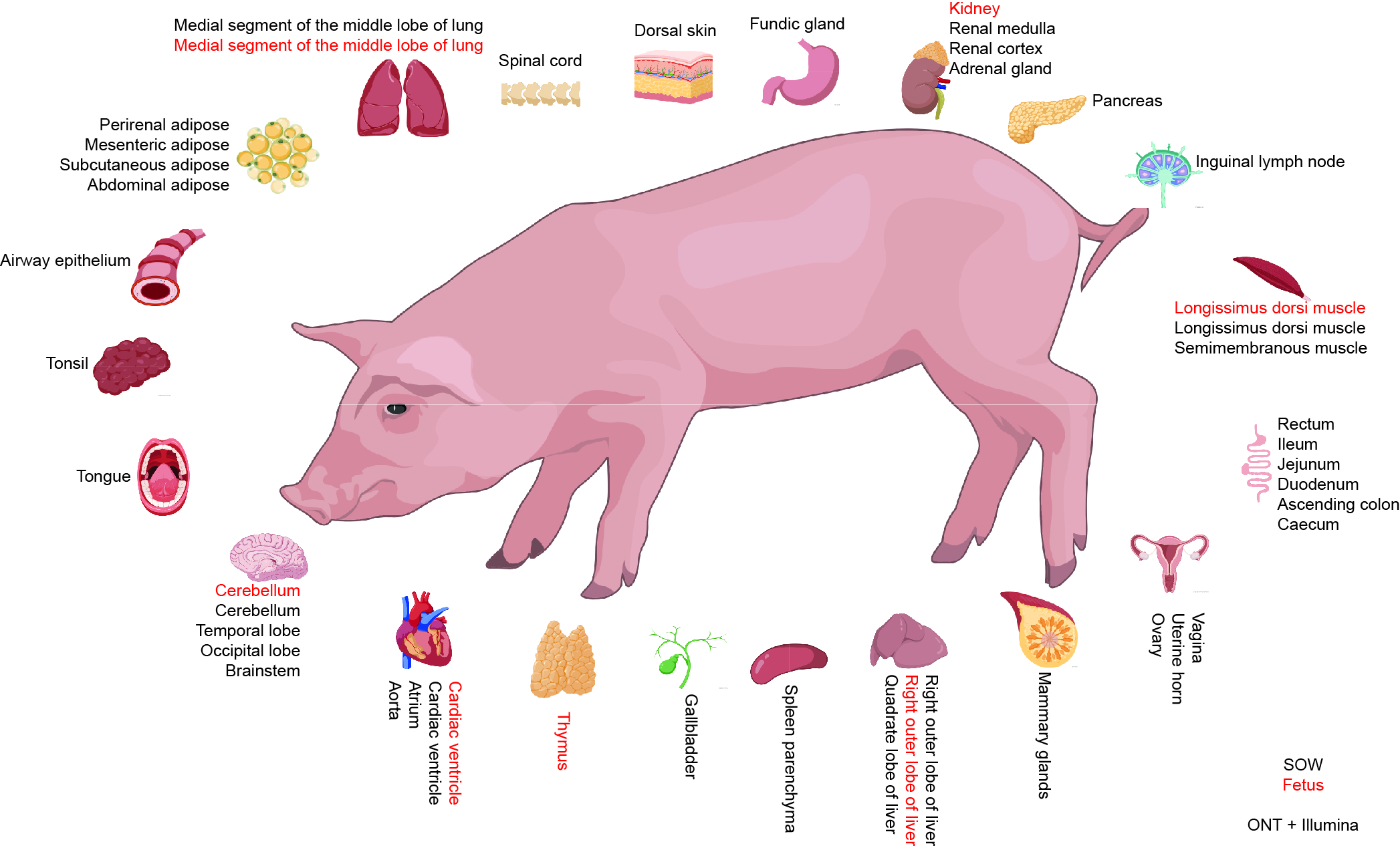


**Supplementary Figure S1.** The pictures of 46 samples that were subjected to ONT DRS and Illumina. The samples in pure black font are from the 2 sows; the samples in pure red font are from the fetus; This figure was Created with BioGDP.com (Jiang et al., 2025).


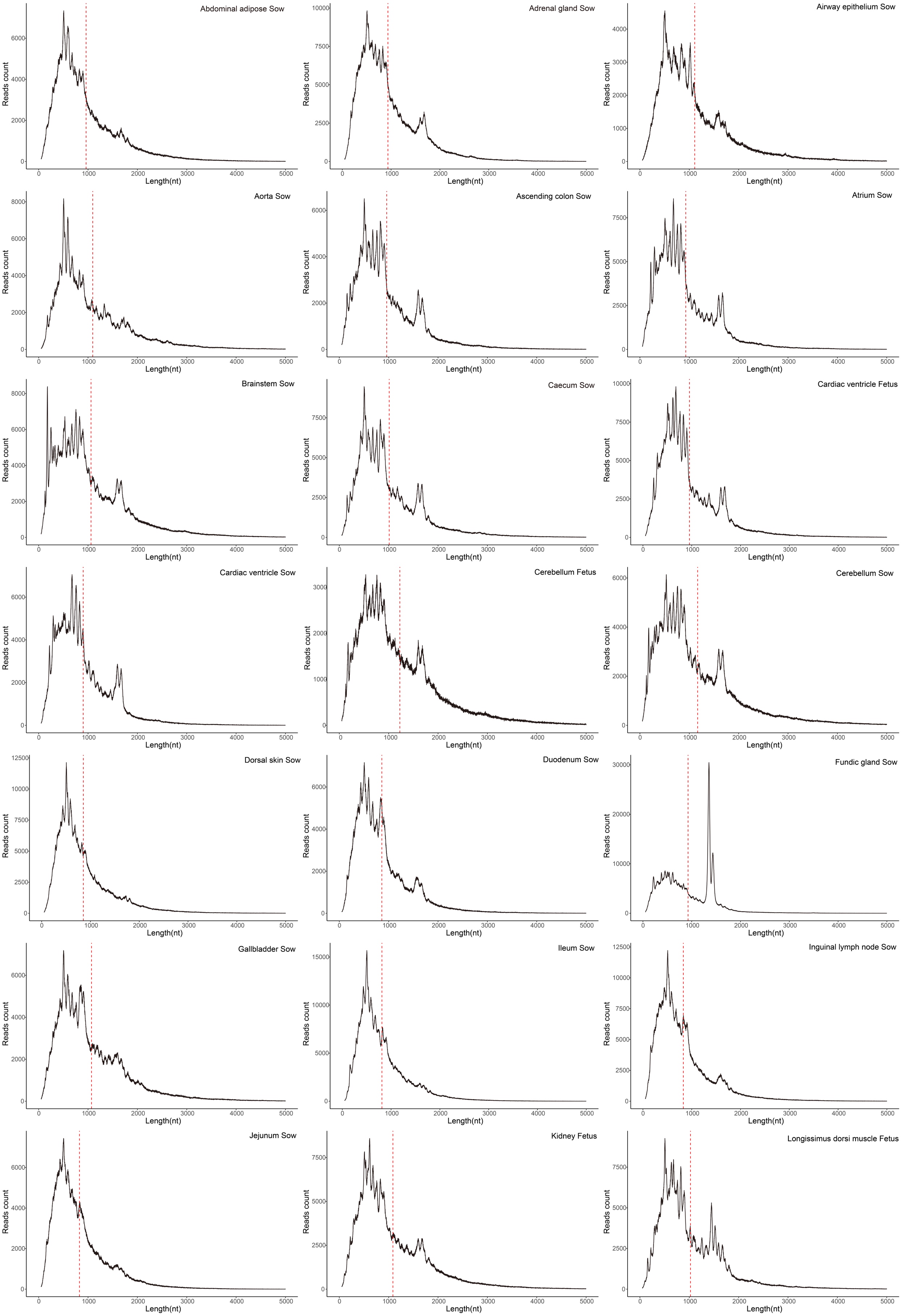


Continued


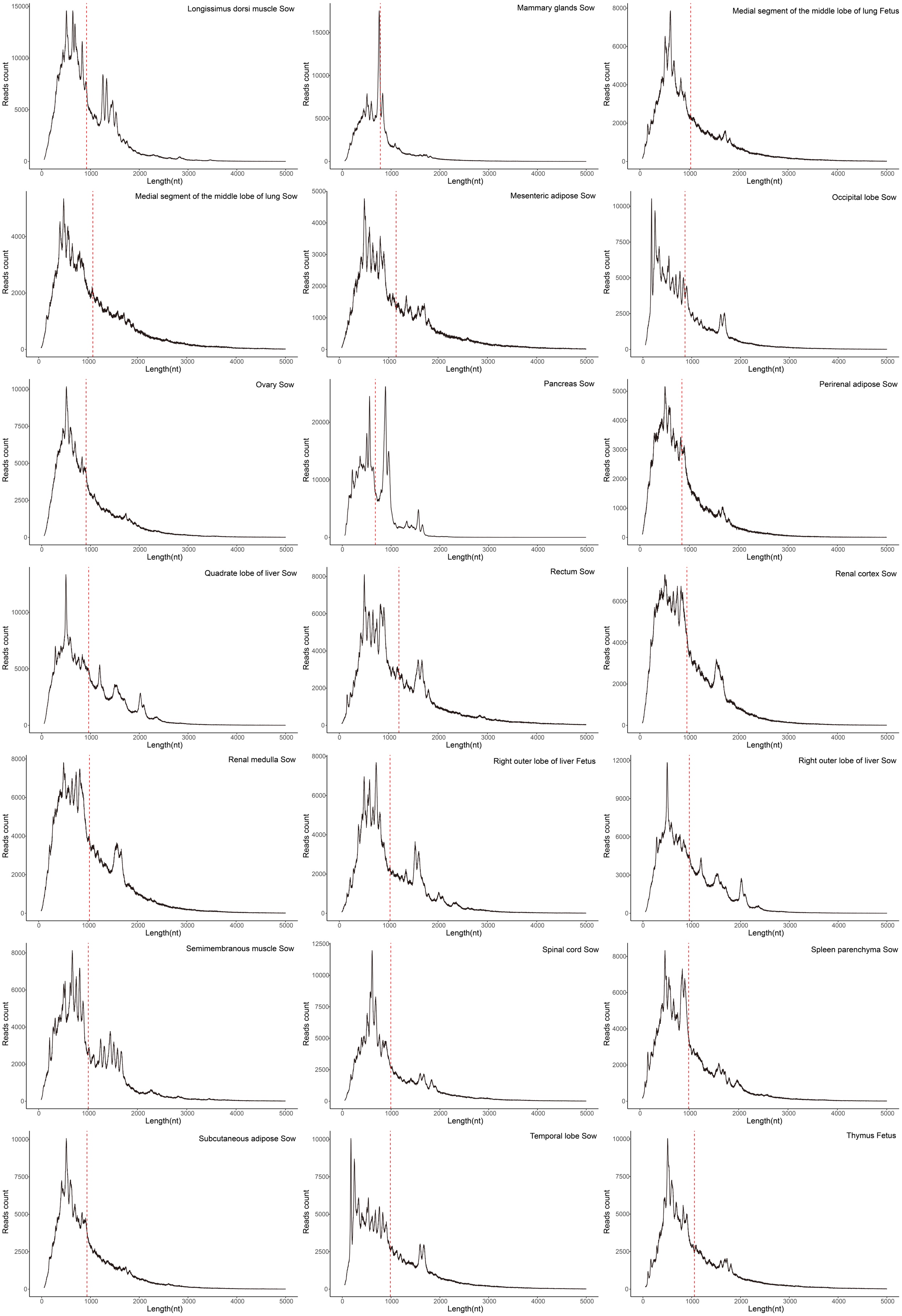


Continued


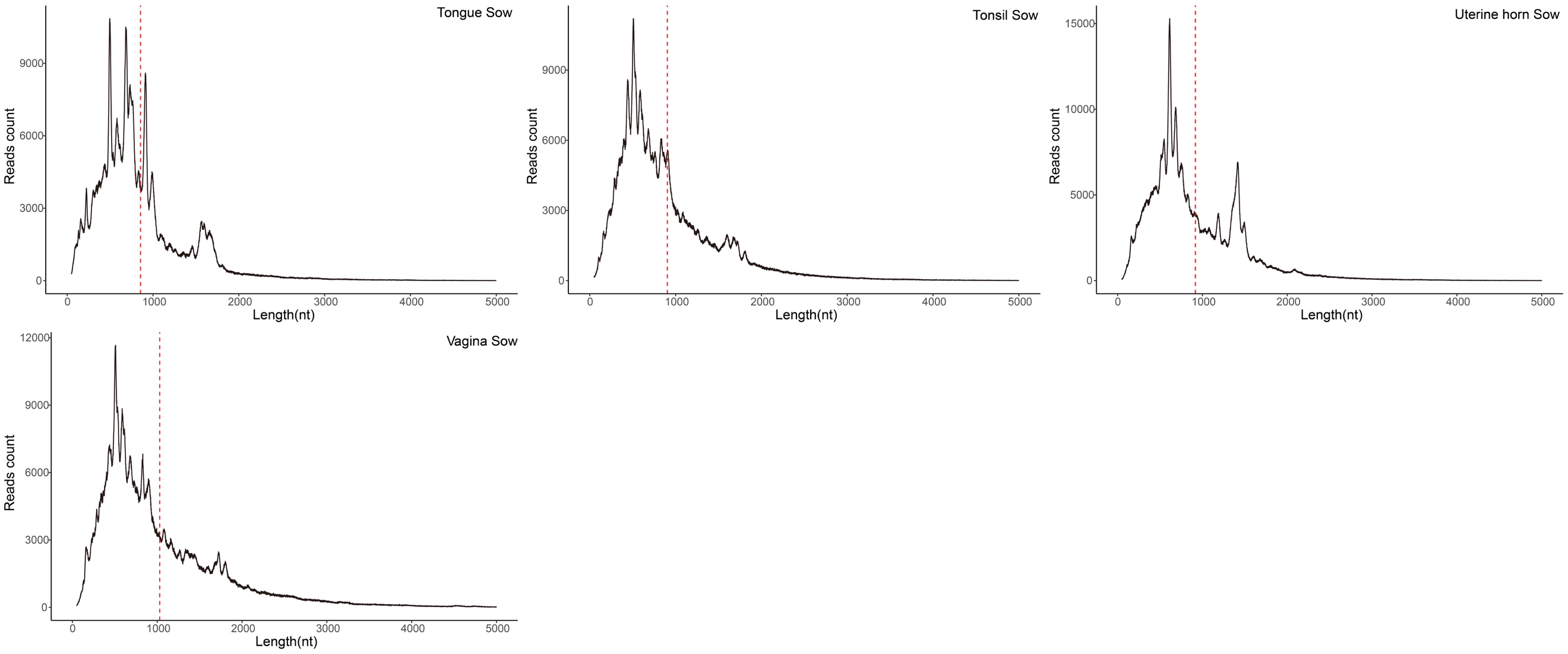


**Supplementary Figure S2.** Read length distribution in the detected tissues. Red dashed lines indicate the mean read length. The X-axis is truncated at 5000 nt.


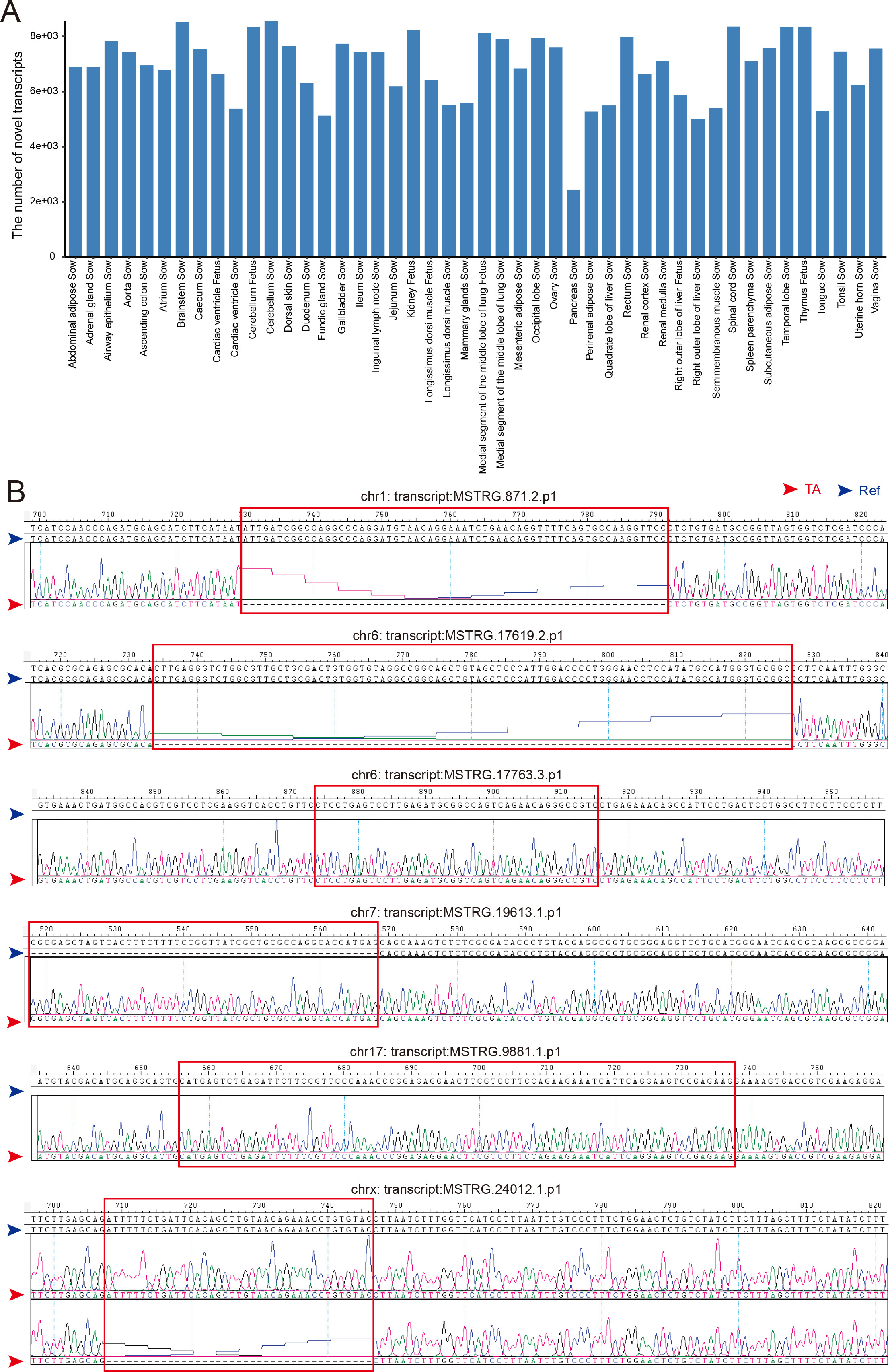


**Supplementary Figure S3**. The number of novel transcripts detected across all samples (A) and sequence alignment of six randomly selected novel isoforms (B).

TA: sequences from TA-cloning sequencing. Ref: reference sequences.


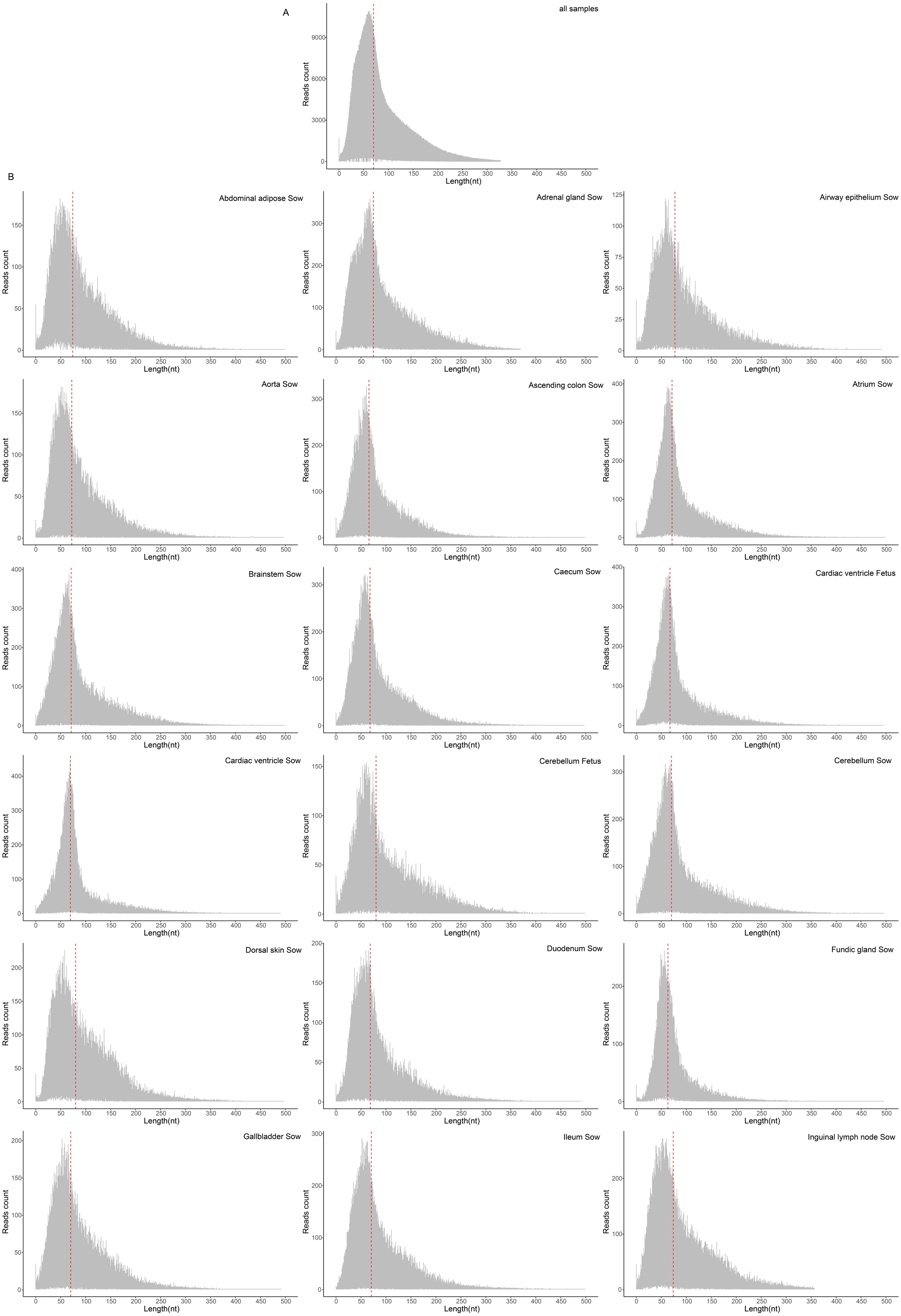


Continued B


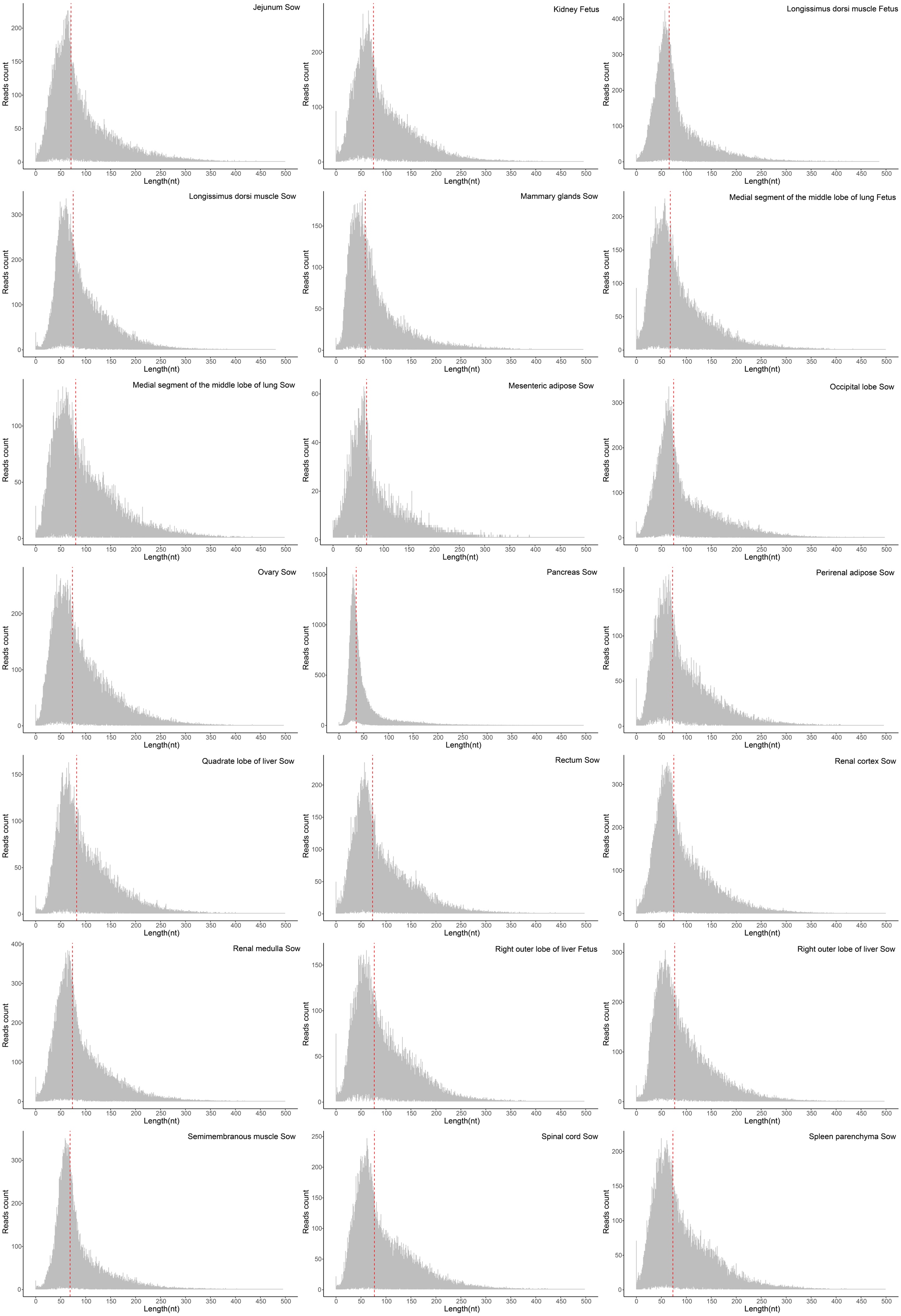


Continued B

**
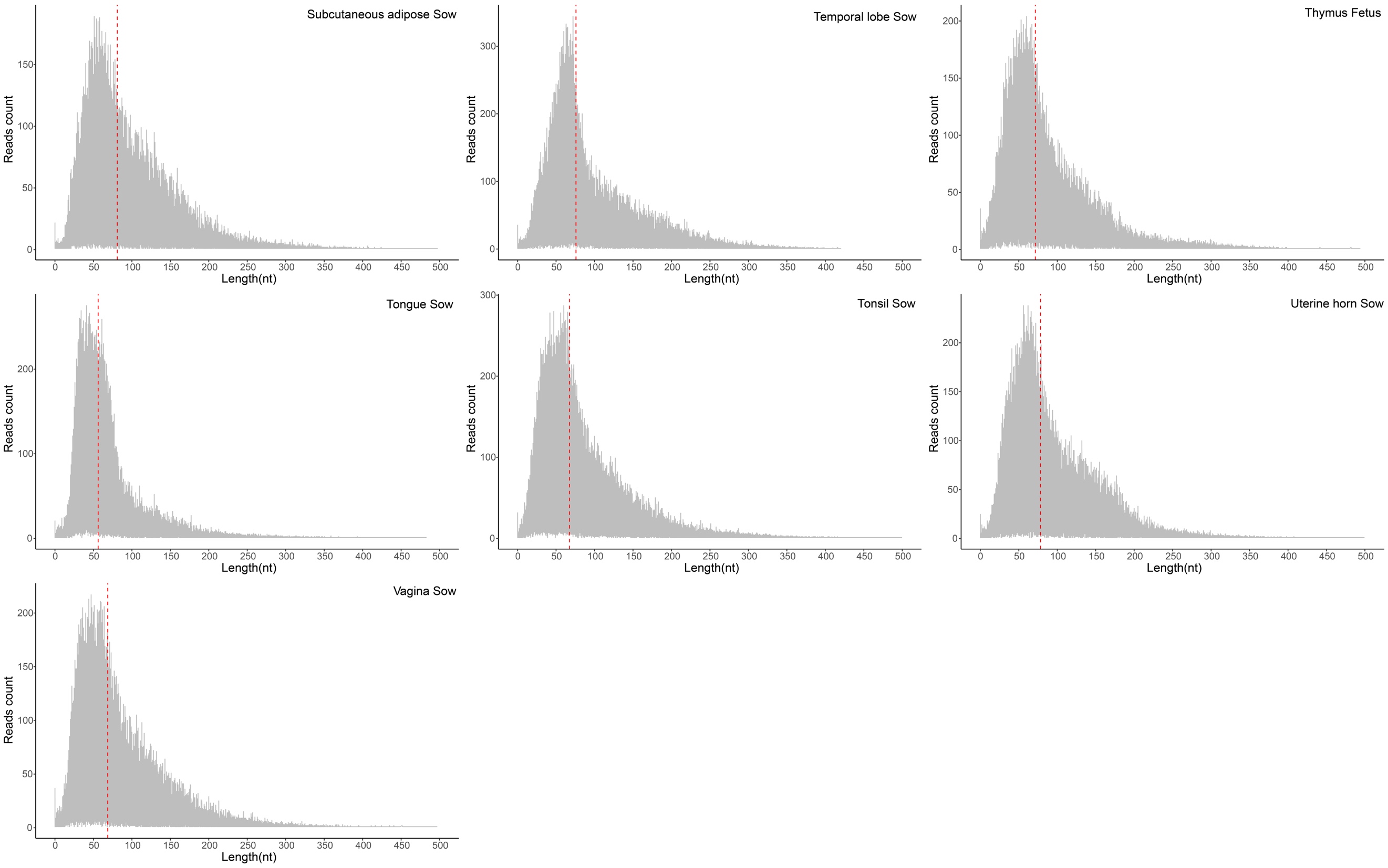
**

**Supplementary Figure S4.** Poly(A) length distribution for all (A) and each (B) detected samples. Red dashed lines indicate the median poly(A) length. X-axis is truncated at 500 bp.

**
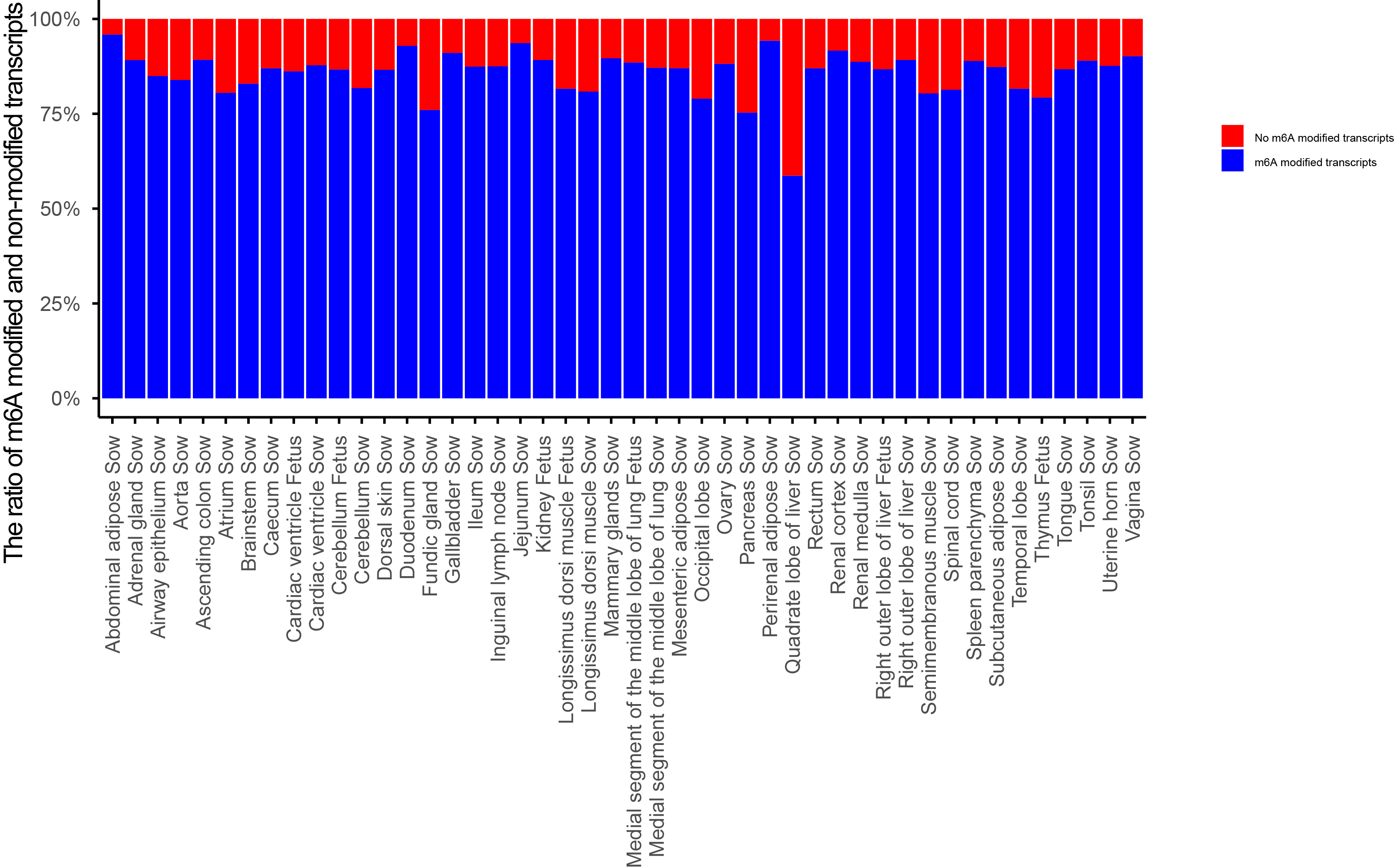
**

**Supplementary Figure S5.** The proportion of m6A modified transcripts in each detected sample.

**
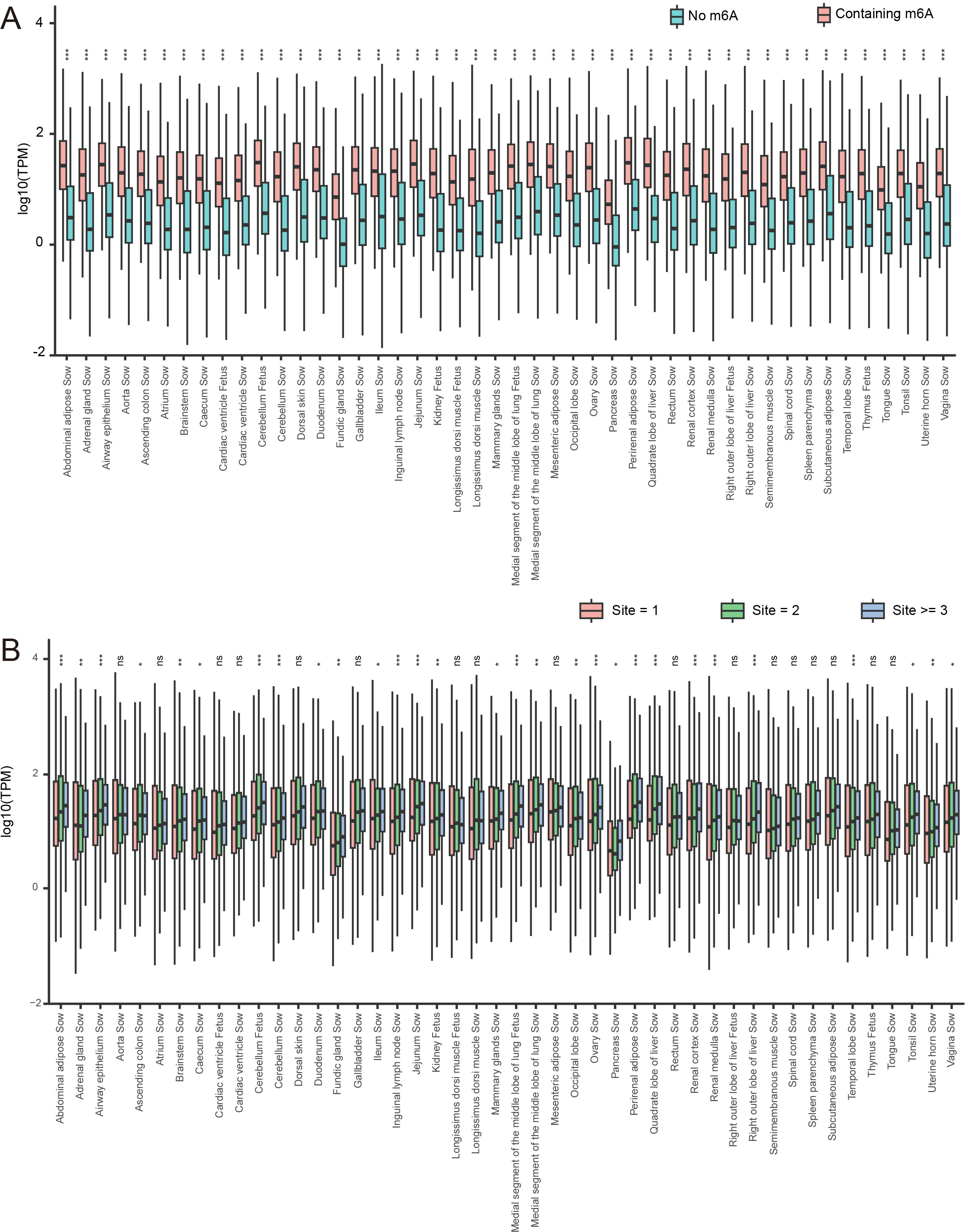
**

**Supplementary Figure S6.** Correlation between m6A methylation levels and transcript expression levels.

A: Comparison of expression levels between transcripts without m6A sites and those containing m6A sites across all samples. B: Comparison of expression levels among transcripts with different numbers of m6A sites. The linear regression was used to assess the correlation between transcript expression levels and m6A methylation. ns: *P* > 0.05; * *P* < 0.05; ** *P* < 0.01; *** *P* < 0.001.

**
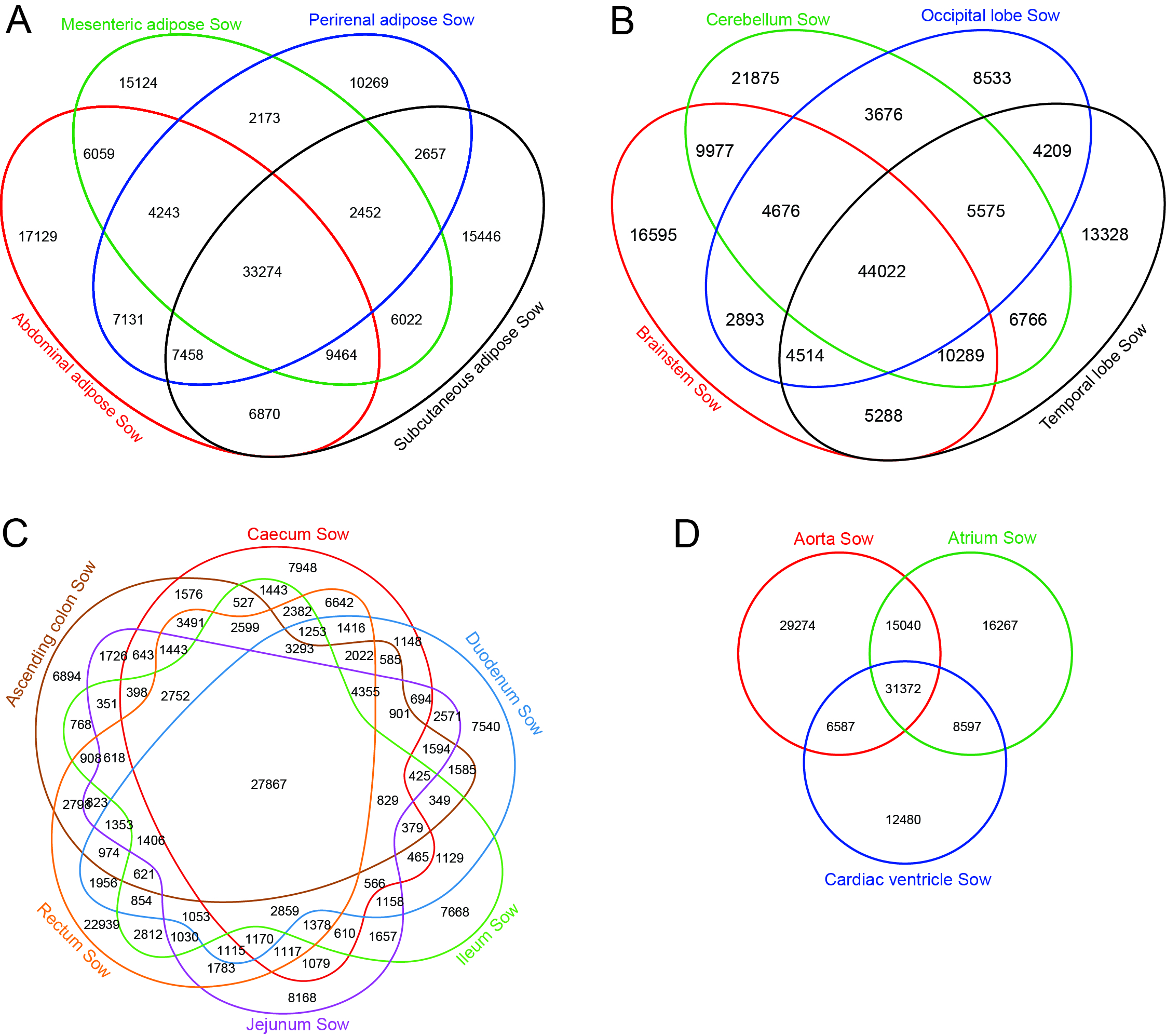
**

**Supplementary Figure S7.** Venn diagrams showing the overlap of m6A sites among distinct anatomical regions of adipose (A), brain (B), intestine (C), and heart (D) tissues.


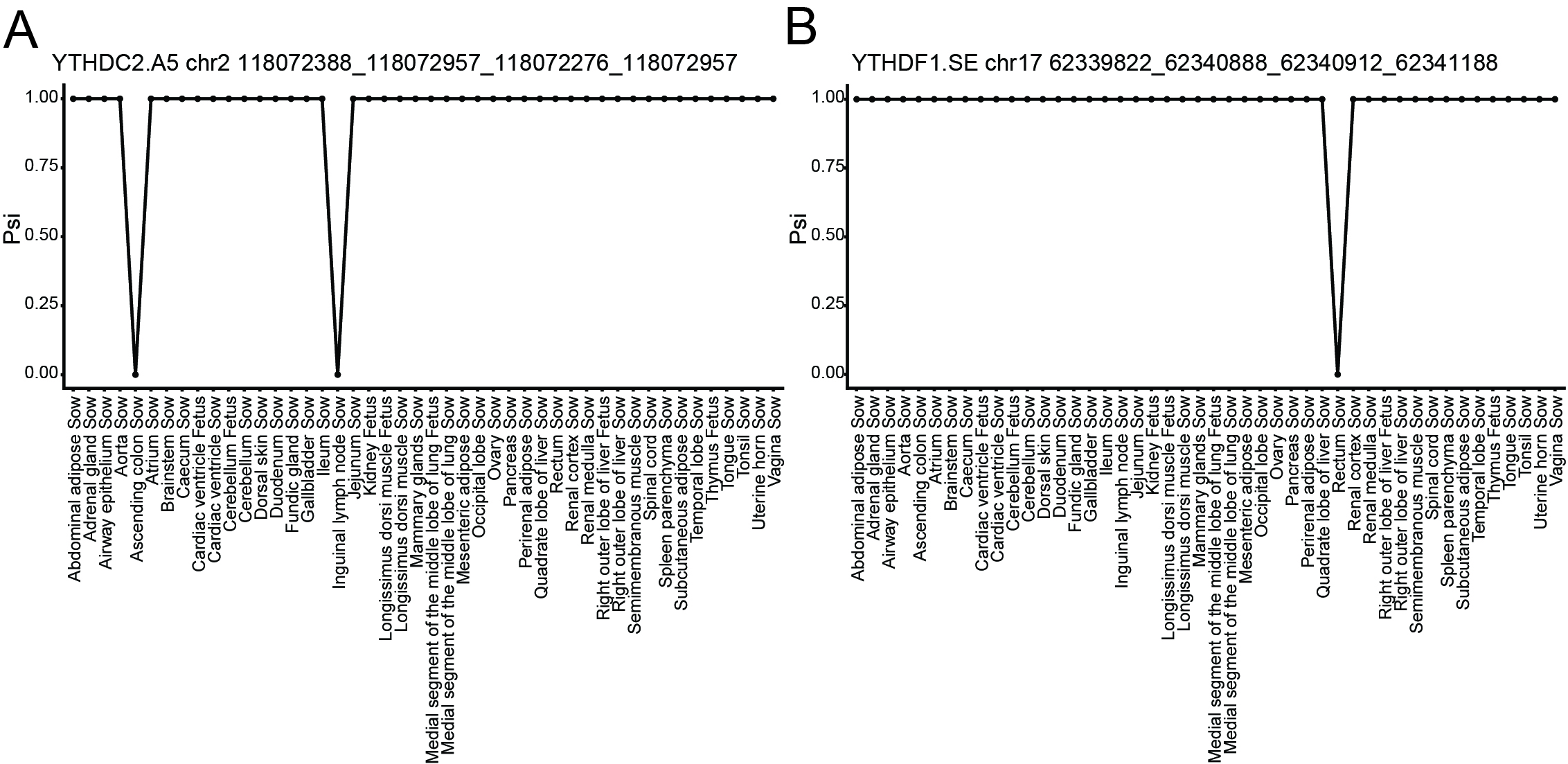


**Supplementary Figure S8.** The Psi values of *YTHDC1* (A) and *YTHDF1*(B) in the detected tissues of sow and fetus. Psi = splice‐in / (splice‐in + splice‐out)

JIANG, S., LI, H., ZHANG, L., MU, W., ZHANG, Y., CHEN, T., WU, J., TANG, H., ZHENG, S., LIU, Y., WU, Y., LUO, X., XIE, Y. & REN, J. 2025. Generic Diagramming Platform (GDP): a comprehensive database of high-quality biomedical graphics*. Nucleic Acids Re*s, **5**3, D1670-d1676.
